# Genome-Wide Analysis of Sorbitol Dehydrogenase (SDH) Genes and Their Differential Expression in Two Sand Pear (*Pyrus pyrifolia*) Fruits

**DOI:** 10.3390/ijms160613065

**Published:** 2015-06-09

**Authors:** Meisong Dai, Zebin Shi, Changjie Xu

**Affiliations:** 1 Zhejiang Provincial Key Laboratory of Horticultural Plant Integrative Biology, State Agriculture Ministry Laboratory of Horticultural Plant Growth, Development and Quality Improvement, Zhejiang University, Zijingang Campus, Hangzhou 310058, China; E-Mail: daimeisong@163.com; 2 Institute of Horticulture, Zhejiang Academy of Agricultural Sciences, Hangzhou 310021, China; E-Mail: shizebinszb@126.com

**Keywords:** sand pear, sorbitol dehydrogenase (*SDH*) gene family, RNA-seq, sugar accumulation, ratio of fructose to sorbitol, fruit development

## Abstract

Through RNA-seq of a mixed fruit sample, fourteen expressed sorbitol dehydrogenase (*SDH*) genes have been identified from sand pear (*Pyrus pyrifolia* Nakai). Comparative phylogenetic analysis of these *PpySDHs* with those from other plants supported the closest relationship of sand pear with Chinese white pear (*P. bretschneideri*). The expression levels varied greatly among members, and the strongest six (*PpySDH2*, *PpySDH4*, *PpySDH8*, *PpySDH12*, *PpySDH13* and *PpySDH14*) accounted for 96% of total transcript abundance of *PpySDHs*. Tissue-specific expression of these six members was observed in nine tissues or organs of sand pear, with the greatest abundance found in functional leaf petioles, followed by the flesh of young fruit. Expression patterns of these six *PpySDH* genes during fruit development were analyzed in two sand pear cultivars, “Cuiguan” and “Cuiyu”. Overall, expression of *PpySDHs* peaked twice, first at the fruitlet stage and again at or near harvest. The transcript abundance of *PpySDHs* was higher in “Cuiguan” than in “Cuiyu”, accompanied by a higher content of sugars and higher ratio of fructose to sorbitol maintained in the former cultivar at harvest. In conclusion, it was suggested that multiple members of the *SDH* gene family are possibly involved in sand pear fruit development and sugar accumulation and may affect both the sugar amount and sugar composition.

## 1. Introduction

Sugar alcohols are widely distributed in nature and perform a central role in metabolism and physiology in various organisms, such as microorganisms, fungi, lower and higher plants. The C_6_ alcohols have been particularly well studied, with mannitol being the most widely distributed, having been found in over 110 higher plant families [1]. Sorbitol, the isomer of mannitol, has been found most abundantly in Rosaceae and proven to be the major form of photosynthate translocated from photosynthesizing source leaves to various sink tissues [2,3]. Sorbitol is also an ideal replacement for sugar for patients suffering from obesity, diabetes, cardiovascular diseases, *etc.* [4].

In Rosaceae fruit trees, when sorbitol reaches the sink tissues, it may be converted into fructose and then used for respiration and biosynthesis of macromolecules, the generation of cell turgor pressure during cell enlargement or act as a signaling molecule controlling various aspects of plant development [5]. In fleshy fruit during maturation and ripening, sorbitol can either be accumulated or be converted into other sugars, thus affecting the sweetness of the fruit, which is a key attribute of fruit quality. The importance of this has been demonstrated in apple, where it has been reported that the fruit quality was notably decreased when sorbitol synthesis in the leaves was silenced [6].

Sorbitol dehydrogenase (SDH, EC 1.1.1.14) is the key enzyme in sorbitol metabolism, which catalyzes the oxidation of sorbitol to fructose in higher plant [7] and in mammals [8]. The corresponding gene is present in various plants, including those, such as *Arabidopsis* [9] and tomato [10], that rarely accumulate sorbitol. However, unlike *Arabidopsis* and tomato and possibly most other plants, which have only one or two *SDH* members in their genomes, the Rosaceae plants apple (*Malus domestica*) and Chinese white pear (*Pyrus bretschneideri*) contain 15 *SDH* members [11]. Recently, the expression patterns and physiological roles of *SDH* members in sink organs have been studied in apple and Japanese pear trees [12,13,14]. However, genome-wide analysis of *SDH* members in relation to pear fruit development and sugar accumulation has not been fully explored.

As the third most important temperate fruit species after grape and apple in the world market, pear is cultivated widely on six continents, especially in China [15]. Commercially, five species of pear are cultivated, including Chinese white pear, sand pear (*P. pyrifolia*), Ussurian pear (*P. ussuriensis*), Xinjiang pear (*P. sinkiangensis*) and European pear (*P. communis*) [16]. Sand pear is widely produced in southern China, as well as in Japan and Korea and is important for regulating market supply because of its early maturation. In this study, the genome-wide analyses of *SDH* genes in five Rosaceae species and another five non-Rosaceae species were conducted. Fourteen *PpySDHs* were identified in sand pear fruit via the RNA-seq method, which showed the closest relationship with Chinese white pear through comparative phylogenetic analysis. The expression of these 14 *PpySDHs* varied greatly among members, and the strongest six accounted for 96% of total transcript abundance of *PpySDHs*. In order to further understand the role of *SDH* in fruit sugar accumulation, the expression patterns of these six members were analyzed in fruit of different developmental stages in “Cuiguan” and “Cuiyu” (Figure S1), two closely-related sand pear cultivars, which showed obvious differences in sugar content. The data obtained could lead to a better understanding of the molecular mechanism for sugar metabolism in pear fruit.

## 2. Results

### 2.1. RNA-Seq of Fruit Tissue of Sand Pear and Identification of PpySDHs

“Cuiyu” sand pear fruit RNA, consisting of an equal mixture or RNA samples from seven developmental stages, was sequenced, with the Chinese white pear genome [11] as a reference for assembly and annotation, to obtain fruit transcriptome data. Approximately 4.5 gigabyte clean bases in 4.6 mega clean reads were obtained, in which the frequency of any nucleotide (N) accounts for only 0.3%; the Q20 (sequencing base calls with an error rate of less than 1%) percentage was over 98%, and 81.42% of clean reads was successfully mapped to the reference genome and assembled into 23,955 distinct genes (Table 1). The number of genes mapped is close to that (24,975 to 28,175) obtained from Chinese white pear fruit transcriptomic analysis previously [17].

**Table 1:** Basic statistics for the RNA-seq data of “Cuiyu” sand pear fruit tissues.

Item	Count/Percentage
Raw reads	48,085,640
Clean reads	46,379,236
Clean bases (bp)	4,505,546,201
Frequency of any nucleotide (N) (%)	0.30
≥Q20 (%)	98.64
Mapped reads	37,763,167
Mapped ratio (%)	81.4
Number of mapped genes	23,955

Expression of 14 *PpySDHs* in the pear genome was detected, but that of *PpySDH7*, corresponding to Pbr032770.1 in Chinese white pear, was not observed.

### 2.2. Sequence Collection and Identification of Sorbitol Dehydrogenase (SDH) Genes

A total of 85 *SDH* homologous genes with complete open reading frames, including 71 from genome sequences and the other 14 from RNA-seq from this study, were identified among 12 plant species. Two sequences were removed because they lacked the functional domain of *SDH* as tested in the Conserved Domains Database (CDD) and Resource Group in NCBI [18]. As a result, 83 genes were identified as *SDH* homologous genes. The number of *SDH* members varied greatly among plant species, e.g., 15 were found in oriental pear specie (*P. bretschneideri*), 22 in occidental pear specie (*P. communis*) and 16 in apple, with from one to four in other species (Table 2).

**Table 2:** Sorbitol dehydrogenase (SDH) homologous genes identified in six Rosaceae species and five non-Rosaceae plant species.

Family Name	Common Name	Species Name	Chromosome Number	Release Version	Genome Gene Number	Identified *SDH* Genes	Gene Name Prefix
Rosaceae	Pear	*Pyrus bretschneideri*	34	NJAU, v1.0	42,341	15	Pbr
		*Pyrus communis*	34	GDR, v1.0	43,419	22	TCONS
	Apple	*Malus domestica*	34	GDR, v1.0	54,921	16	MDP
	Peach	*Prunus persica*	16	GDR, v1.0	27,864	4	EMJ
	Plum	*Prunus mume*	16	NCBI, v1.0	31,390	3	XM
	Strawberry	*Fragaria vesca*	14	GDR, v1.1	32,831	1	mrna
Brassicaceae	*Arabidopsis*	*Arabidopsis thaliana*	10	TAIR, V10.0	27,416	2	AT
Gramineae	Maize	*Zea mays*	20	NCBI, B73_RefGen_v3	39,475	2	GRMZM
Rutaceae	Sweet orange	*Citrus sinensis*	18	GDR, v1.0	29,445	1	Csi
Solanaceae	Tomato	*Solanum lycopersicum*	24	ITAG, V2.3	34,727	1	Solyc
Vitaceae	Grape	*Vitis vinifera*	38	CNS, v1.0	26,346	2	VIT

All identified *SDH* genes belong to the medium chain alcohol dehydrogenase (MDR) superfamily and contained an “l-idonate 5-dehydrogenase” (PLN02702) domain and five other specific features, *i.e.*, the NAD(P) (Nicotinamide adenine dinucleotide (phosphate)) binding site, inhibitor binding site, tetramer interface, catalytic Zn binding site and structural Zn binding site. While 81 out of 83 identified *SDH* genes have a complete PLN02702 domain, that in Pbr032770.1 and TCONS_00034640 is incomplete, possessing only an NAD(P) binding site (Table S1). Interestingly, 20 out of 22 *SDH* homologous genes in *P. communis* have a “threonine dehydrogenase” (COG1063) domain, as well, which is not present in other investigated plants, including other members of the Rosaceae (Table S1).

### 2.3. Phylogenetic Analysis of Predicted SDH Genes

**Figure 1:**
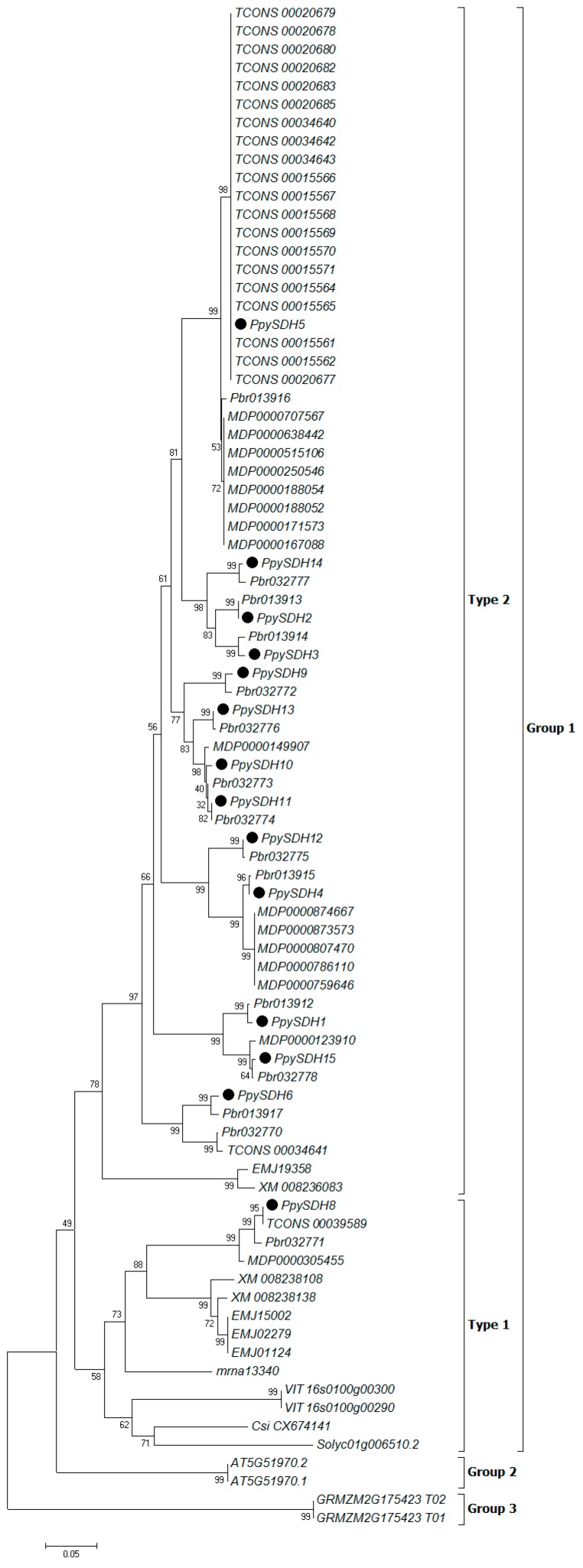
Phylogenetic tree of 83 *SDH* homologous genes from 12 plant species, including 14 *PpySDH* genes (●) identified from the *P. pyrifolia* transcriptome. The phylogenetic tree was generated using the neighbor joining (NJ) method in Molecular Evolutionary Genetics Analysis version 6 (MEGA6) [19]. Numbers given at branch nodes are the bootstrap values of the confidence level, as percentages calculated from 1000 random replications.

In order to investigate phylogenetic relationships and the molecular evolutionary history of the sequences, each member of the plant *SDH* family was used to construct the phylogenetic tree by using the neighbor joining (NJ) method in Molecular Evolutionary Genetics Analysis version 6 (MEGA6) [19]. All *SDH* genes fall into three major clades, with Group 1 containing most of the *SDH* genes from Rosaceae species. Group 2 and Group 3 each have only two genes from a single species (Figure 1).

**Figure 2:**
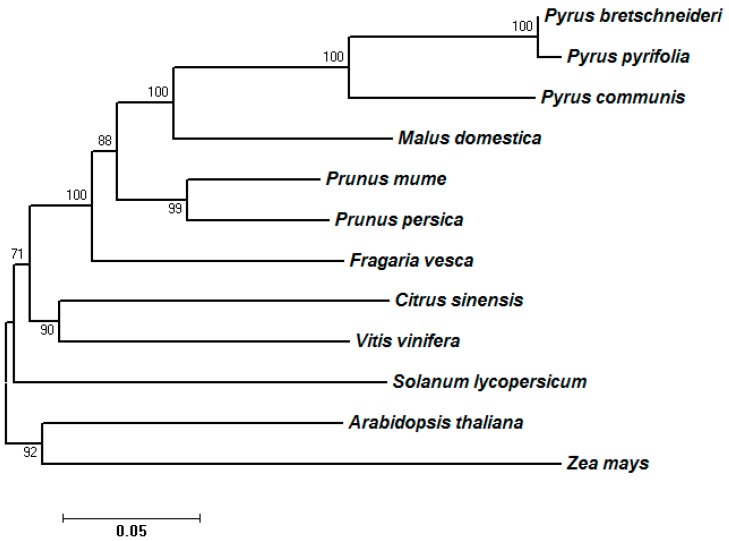
Species tree of seven Rosaceae species and five non-Rosaceae plant species. The protein sequences of different species were first grouped by using OrthoMCL, then sequences in each group were aligned by using Muscle. The tree was constructed by using Molecular Evolutionary Genetics Analysis version 6 (MEGA6) [19] following the ML (maximum likelihood) statistical method and the LG (Le and Gascuel) with Freqs. (+F) amino acid model. The bootstrap method was used to test the phylogeny (500 replications).

From species phylogeny (Figure 2), it can be concluded that the *SDH* genes among *Pyrus* and *Malus* species were closely related, with the closest relationship between *P. pyrifolia* and *P. bretschneideri* genes.

### 2.4. Transcript Abundance of PpySDHs

The transcript abundance of *PpySDHs* varied greatly among members, with that of one member, *PpySDH2*, accounting for over 30% of total abundance and the top six members for over 96%, according to reads per kilo-base per million (RPKM) data (Table 3). These six members were selected for further analysis.

The similarity of nucleotide sequences between *PpySDH* genes and corresponding genes in the reference genome ranged from 91% to 99% (Table S2). The gene structure showed that *PpySDH8* had six exons and five introns; *PpySDH1*, *PpySDH6* and *PpySDH15* had five exons and four introns; *PpySDH5* had four exons and three introns; *PpySDH9*, *PpySDH10* and *PpySDH11* had three exons and two introns; the remaining six *PpySDH* members had two exons and one intron (Figure S2).

**Table 3:** Expression of *SDH* genes in sand pear fruit. Values shown are TPM (transcripts per million) and RPKM (reads per kilo-base per million). Lines marked with grey background denote the top six most strongly-expressed members.

Gene Name	Corresponding Gene in Reference Genome	TPM	RPKM	Percentage in Total RPKM (%)
*PpySDH1*	Pbr013912.1	8147.01	7244.58	0.93
*PpySDH2*	Pbr013913.1	270,655.77	240,675.52	30.99
*PpySDH3*	Pbr013914.1	7402.56	6582.59	0.85
*PpySDH4*	Pbr013915.1	125,806.21	111,870.79	14.40
*PpySDH5*	Pbr013916.1	3874.83	3445.62	0.44
*PpySDH6*	Pbr013917.1	360.01	320.13	0.04
*PpySDH7*	Pbr032770.1	0	0	0.00
*PpySDH8*	Pbr032771.1	41,652.83	37,039	4.77
*PpySDH9*	Pbr032772.1	642.55	571.37	0.07
*PpySDH10*	Pbr032773.1	4841.99	4305.65	0.55
*PpySDH11*	Pbr032774.1	4253.54	3782.38	0.49
*PpySDH12*	Pbr032775.1	174,199.23	154,903.37	19.95
*PpySDH13*	Pbr032776.1	21,448.95	19,073.08	2.46
*PpySDH14*	Pbr032777.1	207,687.56	184,682.23	23.78
*PpySDH15*	Pbr032778.1	2408.97	2142.13	0.28

### 2.5. Tissue-Specific Expression of Six Selected PpySDHs

**Figure 3:**
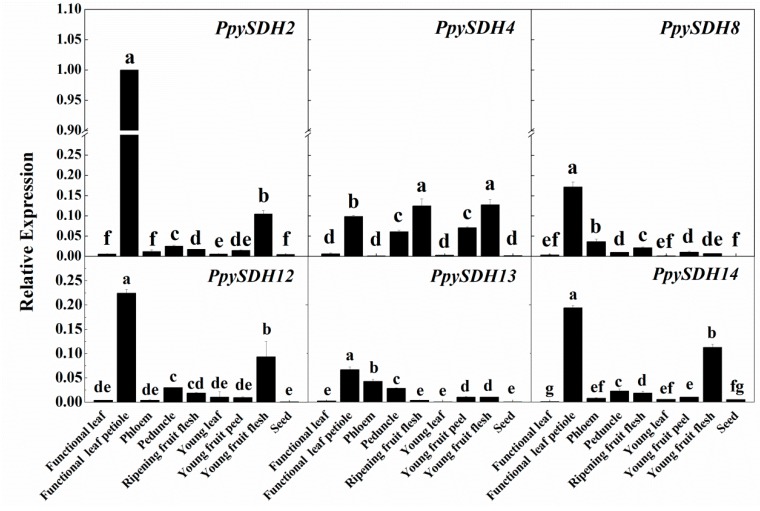
Relative expression levels of six selected *PpySDH* genes in nine tissues of sand pear (*P. pyrifolia* Nakai. cv. Cuiyu). The expression of each gene was first normalized against *18S rRNA* (internal control) by using the 2^−Δ*C*t^ method, and then, the strongest expression, *PpySDH2* in functional leaf petiole in this figure, served as a calibrator (1.0); expression of the remaining genes was expressed as a multiple of the calibrator. Error bars indicate the standard error (SE) from three biological replicates. Different letters within each chart indicate means that are significantly different (*p* ≤ 0.05).

Tissue-specific expression patterns of the above-mentioned six most strongly-expressed *PpySDHs* were analyzed with nine tissues or organs of “Cuiyu” sand pear. The strongest expression was observed for *PpySDH2* in functional leaf petiole, followed by several other members in leaf petiole tissue (Figure 3). Expression of four members, *PpySDH2*, *PpySDH4*, *PpySDH12* and *PpySDH14*, was also high in young flesh tissues. In mature fruit flesh, *PpySDH4* was the main expressed member. Expression was relatively low in functional leaves, young leaves and seeds for all six members analyzed (Figure 3).

### 2.6. Expression of PpySDHs during Fruit Development

**Figure 4:**
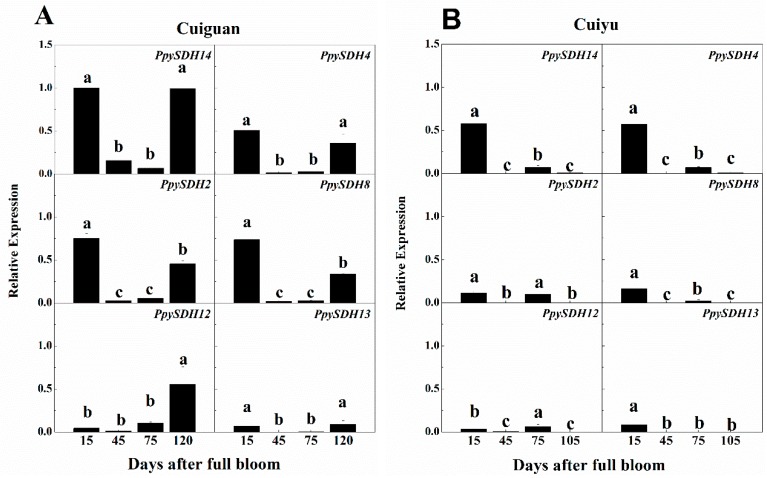
Relative expression levels of six selected *PpySDH* genes during fruit development of “Cuiguan” (**A**) and “Cuiyu” (**B**). The expression of each gene was normalized against *18S* rRNA (internal control) by using the 2^−Δ*C*t^ method, and then, that with the strongest expression, *PpySDH14* of “Cuiguan” at 15 days after full bloom in this figure, served as a calibrator (1.0); the expression of the remaining genes, including all analyzed members from “Cuiyu”, were expressed as a multiple of the calibrator. Error bars indicate the standard error (SE) from three biological replicates. Different letters within each chart indicate means that are significantly different (*p* ≤ 0.05).

Overall, the relative expression level of *SDH* members was higher in “Cuiguan” than in “Cuiyu” (Figure 4). The normalized relative expression value of the six *SDH* members at all stages was 6.41 in “Cuiguan” and 4.37 in “Cuiyu”.

The six *SDH* members in “Cuiguan” could be divided into two groups based on the expression pattern during fruit development (Figure 4A). One group contained five members, *PpySDH2*, *PpySDH4*, *PpySDH8*, *PpySDH13* and *PpySDH14*, which were strongly expressed at both young (15 days after full bloom (DAFB)) and ripening stages (120 DAFB) and remained at a lower level during the middle developmental stage (45 and 75 DAFB). The other group contained only one member, *PpySDH12*, which remained expressed at a low level until fruit ripening. In “Cuiyu” pear fruit (Figure 4B), the relative expression levels of all six members were higher at 15 and 75 DAFB, but remained lower at the middle developmental stage (45 DAFB) and at harvest (105 DAFB).

### 2.7. Changes in Sorbitol Content in Flesh during Fruit Development

In order to further elucidate the relationship between the expression pattern of the six *PpySDH* members and sugar accumulation during fruit development, seasonal changes in sorbitol content in the fruit of two cultivars were conducted.

For ripe fruit, no significant differences in average fruit weight and firmness of fruit flesh were observed between “Cuiguan” and “Cuiyu”; however, the total soluble solids content was remarkably higher in “Cuiguan” (Table 4).

**Table 4:** Physiology index of two sand pear cultivars, “Cuiguan” and “Cuiyu”.

Cultivar	Commercial Harvest Date (D/M)	Average Fruit Weight (g)	Soluble Solids Content (%)	Firmness of Fruit Flesh (kg·cm^−2^)
Cuiguan	27/7	302.5 ± 6.7 ^a^	13.08 ± 0.49 ^a^	2.65 ± 0.65 ^a^
Cuiyu	12/7	295.0 ± 11.1 ^a^	10.77 ± 0.80 ^b^	2.23 ± 0.32 ^a^

Different letters within each column indicate means that are significantly different (*p* ≤ 0.05).

**Figure 5:**
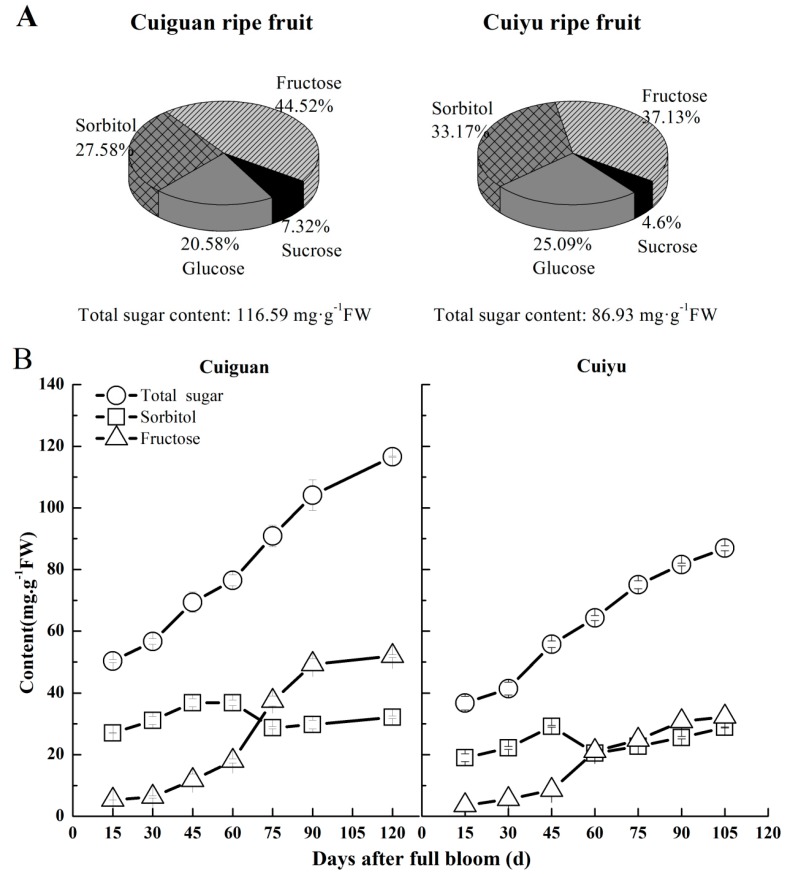
Sugar content and composition in ripe fruit (**A**) and changes in content of total sugars (○), sorbitol (□) and fructose (∆) during fruit development (**B**) in “Cuiguan” and “Cuiyu” pears. Error bars indicate the standard error (SE) from three biological replicates.

The content of total sugars was 34% higher in “Cuiguan” than “Cuiyu”. Fructose, sorbitol, glucose and sucrose were the main soluble sugars in ripened pear fruit in both cultivars; however, “Cuiguan” was characterized by a higher percentage of fructose and lower percentage of sorbitol (Figure 5A). During fruit development, total sugar levels continued to increase with somewhat different patterns between two cultivars (Figure 5B). Fructose in particular increased to a greater amount in “Cuiguan”, reaching 51.91 mg/g FW (fresh weight), 61% higher than that in “Cuiyu” (Figure 5B).

**Figure 6:**
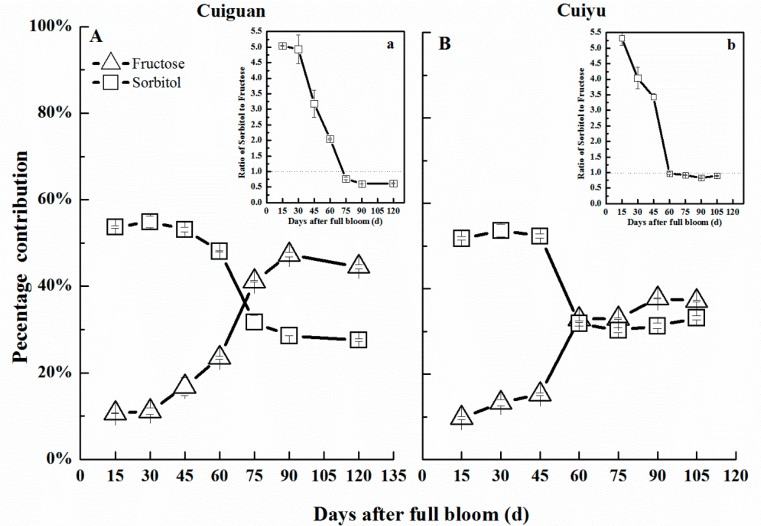
Percentage sorbitol (□) and fructose (∆) content in total sugars (**A**,**B**) and the ratio of sorbitol to fructose (**a**,**b**) during fruit development of two cultivars, “Cuiguan” (**A**,**a**) and “Cuiyu” (**B**,**b**). Error bars indicated standard error (SE) from three biological replicates.

The percentage contribution of sorbitol was high, representing over half of the total sugars, during early fruit development, decreased sharply during the fruit enlargement stage and remained stable during the late developmental stage, with values slightly lower in “Cuiguan” (Figure 6). Fructose accounted for about 10% of total sugars in fruitlets (15 DAFB) and increased during development until ripening (Figure 6). The percentage of fructose in ripe fruit was higher, around 45%, in “Cuiguan” than in “Cuiyu”, which was around 37% (Figure 5A,B). As a result, the ratio of sorbitol to fructose was much lower in “Cuiguan” during late fruit development (Figure 6).

## 3. Discussion

Over recent years, multiple members of the *SDH* gene family have been reported in Rosaceae plants [12,13,14,20], as well as in some other plants, such as grape and sweet orange [21]. A total of 15 *SDH* genes were found in the genome of Chinese white pear [11]. In this study, 14 of 15 *SDH* homologous genes to Chinese white pear were found to be expressed in sand pear fruit tissues based on RNA-seq data, and the nucleotide sequences of the obtained 14 members were over 91% identical to that of Chinese white pear (Table S2). These results, together with the data from comparative phylogenetic analysis (Figure 1 and Figure 2), indicated that sand pear is a very close relative to Chinese white pear, which supports the taxonomic theory that the cultivars of sand pear, Chinese white pear and Japanese pear may have originated from the common progenitor, sand pear [16].

All 14 expressed *PpySDHs* belonged to the MDR (medium-chain dehydrogenase/reductase) superfamily with the features of containing a NADP binding site, an inhibitor binding site, a tetramer interface, a catalytic Zn binding site and a structural Zn binding site in the corresponding proteins [22]. However, for Pbr032770.1, with counterpart *PpySDH7* in sand pear, which was not expressed in fruit tissues, the predicted protein only had an NAD(P) binding site, with the remaining four functional domains absent (Table S1). It can be suggested that potential deletion events might have occurred during the evolution of the *SDH* gene family in *Pyrus*, which is quite common in Rosaceae [11]. Apart from the presence of the “l-idonate 5-dehydrogenase” domain in all pear SDH, the occurrence of another domain, the “threonine dehydrogenase” domain, was also observed in 20 out of 22 SDH in *P. communis* (Table S1). Interestingly, this “threonine dehydrogenase” domain is not present in SDH of other investigated plants, even other Rosaceae plants (Table S1). However, when searching for homologous nucleotide sequences of this domain in the *P. bretschneideri* genome, highly homologous sequences could be obtained. Therefore, the unique presence of the “threonine dehydrogenase” domain in SDH of *P. communis* can be the result of a distinctive pre-mRNA processing where the DNA region corresponding to this domain was kept as an exon rather than an intron. The reasons for such a distinctive pre-mRNA processing and the metabolic roles and anabolic activities of a bi-domain remain unclear and are worthy of further study.

Expression patterns of *PpySDHs* varied greatly among different gene family members. In this study, genome-wide expression analysis was carried out with six genes identified as the most strongly-expressed members, *PpySDH2*, *PpySDH4*, *PpySDH8*, *PpySDH12*, *PpySDH13* and *PpySDH14* (Table 3). The expression of some of these members has also been studied in some previous reports. For example, members *PpySDH2*, *PpySDH4*, *PpySDH12* and *PpySDH14* were expressed in pear buds and were suggested to be a requisite for bud growth and development [14]. Expression of *PpySDH8* in leaves was also reported [20]. However, the detailed information on the contribution of members to the individual tissue or organ had not been previously studied.

Expression patterns of *PpySDHs* varied greatly in different tissue or organ types (Figure 3). These data were generally consistent with the previous reports on tissue-specific regulation of *SDH* expression in apple (fruit, young and old leaves, stems, roots, seeds, *etc.*) [23,24,25] and pear (leaves, buds, fruits, *etc.*) [14,20,26]. All six of the most strongly-expressed *PpySDHs* showed the highest transcript levels in functional leaf petiole, followed by phloem (Figure 3), which might help to unload more photosynthates from source leaves to sinks [27]. *SDH* has also been found widely expressed in both source and sink organs in some non-Rosaceae species. For instance, the highest expression level was found in the seed of *Arabidopsis thaliana*, followed by leaves, roots, *etc.*, and the lowest in siliques [9]. In maize, *SDH* was only activated in kernel and endosperm, and undetectable levels of activity were found in tassel, leaf, stem and root [28]. *SDH* transcription was also found in strawberry fruit [29]. These indicated the ubiquitous distribution and fundamental function of *SDH*s in the plant kingdom.

Sugars are major components of fruits. It has been well-known that total sugar content in fruits was primarily genotype dependent [30]. As revealed in this study, divergence in sugar accumulation was observed between “Cuiguan” and “Cuiyu”, though these two tested cultivars were actually quite closely related, with “Cuiguan” being the male parent of “Cuiyu” [31]. Mining the reasons for differential sugar accumulation among plant species and cultivars is an interesting and important topic for fruit scientists. Recently, it has been generally accepted that the sink strength plays as important or maybe more important roles than the source in fruit sugar accumulation, as it has been suggested that the movement of photosynthates into the pear fruit was independent of export from the source leaf, but determined by the sink strength [32]. The sink strength has been considered as a product of sink size and sink activity [33]. In this study, because the two cultivars tested have similar fruit growth rates and fruit sizes (Table 4, Figure S3), their sink size can be regarded as similar. Therefore, it can be inferred that the stronger sink strength could be the primary factor for the higher accumulation of sugars in “Cuiguan” fruit. The expression level of *PpySDHs* was several times higher in “Cuiguan” than in “Cuiyu” (Figure 4), and the possibility that this contributed to a stronger sink strength in “Cuiguan” is worthy of further study. A higher relative expression of *PpySDH2*, *PpySDH4* and *PpySDH14* was found during fruit development of both cultivars (Table 3, Figure 4), and therefore, these members can be preferentially selected to be investigated to determine whether they play important roles in regulating sugar accumulation and compositions in pear fruits.

Sugar composition in fruits is also genotype dependent. Sucrose is the main sugar in fruits of peach, apricot, pineapple, muskmelon, *etc.*, while hexoses are the main ones in fruits of grape, citrus, kiwifruit, papaya, sweet cherry, *etc.* [24,34,35,36]. In sand pears “Cuiguan” and “Cuiyu”, the most abundant sugar molecules were sorbitol and fructose (each of which made up of around one third of the total sugars) (Figure 5). The sorbitol percentage was similar to or higher than in some other pear cultivars [37]. Interestingly, these differences in sugar composition also contributed greatly to sweetness, as common sugars have been given a relative sweetness of sucrose 100, fructose 175, glucose 70 and sorbitol 40 [38]. As observed in this study, ripe fruit of “Cuiguan” had a higher content of both total soluble sugars and fructose, as well as a higher percentage of fructose than “Cuiyu” (Figure 5), which can explain why “Cuiguan” fruits taste sweeter than “Cuiyu”. Since SDH catalyzes the oxidation of sorbitol to fructose, the higher percentage of fructose in “Cuiguan” ripe fruit may be at least partially related to the stronger expression of *PpySDHs*. This needs to be further investigated, and if SDH does play an important role in regulating sugar accumulation and composition, then manipulation of *SDH* can be an additional way to make fruit sweeter.

## 4. Experimental Section

### 4.1. Plant Materials

Two sand pear cultivars (*P. pyrifolia* Nakai cv. Cuiguan and Cuiyu) grown at Haining City, Zhejiang Province, R. P. China, were used in this study. “Cuiguan” is a major commercial sand pear cultivar grown along the Yangtze River basin in China [15], and “Cuiyu” is a newly-released sand pear cultivar with the characteristics of early-ripening and good fruit appearance, but a less sweet taste [31]. “Cuiguan” is also the male parent of “Cuiyu” [31].

“Cuiyu” pear fruit at seven developmental stages (15, 30, 45, 60, 75, 90 and 105 DAFB) was collected in 2013, with at least six fruit for each stage. The fruit was subjected to further RNA extraction, and equivalent amounts of total RNA from each stage were mixed for RNA-seq.

At 54 DAFB, about half way through the development of “Cuiyu” fruit, the fruit flesh, fruit peel, seed, phloem, young leaf, functional leaf, functional leaf petiole and peduncle were collected. For analysis of fruit soluble sugar content and other physiological indices, a total of 7 samples of each cultivar were collected regularly from 15 days after full bloom (DAFB) until harvest. All samples were separated and frozen immediately in liquid N_2_, then stored at −70 °C until further use. Each sampling consisted of three biological replicates, each with six fruits or 20 grams of other tissues.

### 4.2. Measurement of Soluble Solids Content (SSC) and Fruit Firmness

Soluble solids content (SSC) was measured by Pocket Refractometer PAL-1 (ATAGO Co., Ltd., Tokyo, Japan), and fruit firmness was measured by GY-1 hand sclerometer (TOP Instrument, Hangzhou, China).

### 4.3. HPLC Analysis of Sugar Content

HPLC analysis of sugar content was carried out according to [39] with slight modification. Briefly, approximately 1 g of frozen powder was extracted with 80% ethanol at 80 °C for 40 min. The sugar solution was collected by 10 min centrifugation at 2500× *g*, and the residues were re-extracted twice. The sugar solutions were combined and brought to a final volume of 25 mL, and then 3.5 mL of solution were taken and vacuum evaporated. The residue was first dissolved with 1 mL of distilled water, then filtered by using an Oasis^®^ HLB (1 cc, 30 mg, Waters China Ltd., Hong Kong, China) column and aqueous membrane syringe filter (Φ0.22 µm). A 20-µL aliquot was passed through a Sugar-Pak™ 1 chromatographic column (6.5 × 300 mm) and analyzed with an HPLC system (Waters 1525 pump, Waters 717 plus auto sampler, Waters 2414 detector) (Waters China Ltd., Hong Kong, China). Breeze™ control software Version 3.30 (Waters China Ltd., Hong Kong, China) was used for HPLC control and data analysis. Standard dilutions with known concentrations of glucose, fructose, sucrose and sorbitol (Sigma-Aldrich Co. LLC., St. Louis, MO, USA) were used as references. The content of total sugar was taken as the sum of glucose, fructose, sucrose and sorbitol content.

### 4.4. RNA Isolation and RNA-Seq Analysis

Total RNA was extracted from frozen powder by using PureLink^®^ Plant RNA Reagent (Ambion^®^, Thermo Fisher Scientific Inc., Waltham, MA, USA) and pretreated with DNase I using TURBO DNA-free™ Kit (Thermo Fisher Scientific Inc.), according to the manufacturer’s protocol. RNA integrity was electrophoretically verified with ethidium bromide staining and purity by checking that the A260/A280 absorption ratio was between 1.9 and 2.1.

Equal amounts of total RNA from fruit at each developmental stage were mixed for RNA-seq, which was carried out by Shanghai Majorbio Bio-Pharm Technology Co., Ltd. (Shanghai, China). In summary, RNA-seq libraries were constructed using an Illumina standard mRNA-Seq Prep Kit (TruSeq RNA and DNA Sample Preparation Kits Version 2, Illumina, Inc., San Diego, CA, USA), then small RNAs ligated with adaptors were used to run RT-PCR during the production of sequencing libraries, and finally, the products were purified and sequenced on an Illumina Hi-Seq 2000 Sequencer.

The transcriptome was aligned and mapped to the Chinese white pear reference genome [11] with TopHat [40]. The relative abundance of transcripts were estimated with the Cufflinks software [41].

### 4.5. Construction of Phylogenetic Tree

The full-length coding sequences of the 15 *SDH* genes in *P. bretschneideri* were used to search homologous sequences via OrthoMCL software [42] in five fully-sequenced genomes of Rosaceae species, pear (*P. communis*), apple (*Malus domestica*), peach (*Prunus persica*), plum (*Prunus mume*) and strawberry (*Fragaria vesca*), as well as five non-Rosaceae plant species, *Arabidopsis* (*Arabidopsis thaliana*), tomato (*Solanum lycopersicum*), maize (*Zea mays*), sweet orange (*Citrus sinensis*) and grape (*Vitis vinifera*). The database versions used in this study are listed in Table 2.

Multiple alignments of the predicted amino acid sequences were used to construct the phylogenetic tree in order to investigate phylogenetic relationships and the molecular evolutionary history of the sequences by using the neighbor joining (NJ) method in MEGA6 [19]. A Bootstrap test was set as 1000 to test the confidence of the tree.

### 4.6. Quantitative Reverse Transcriptase PCR (qRT-PCR) and Data Analysis

For qRT-PCR analysis, a PrimeScript^®^ II Transcriptase (TaKaRa Bio Inc., Kusatsu, Shiga, Japan) was used to synthesize the first-strand cDNA from 1 μg of total RNA in a final volume of 20 μL, containing 4 μL of 5× PrimerScript^®^ buffer, 1 μL of Oligo (dT)_18_ primers, 1 μL of dNTP mixture (10 mM each), 0.5 μL of RNase inhibitor (40 U/μL) and 0.5 μL of PrimeScript^®^ II transcriptase (200 U/μL) (TaKaRa, Bio Inc., Kusatsu, Shiga, Japan). The cDNA samples were amplified with gene-specific primers (designed based on genome information and the sequences listed in Table 5) by using LightCycler^®^ 480 SYBR Green I Master (Roche Diagnostics, West Sussex, UK). The melting curve analysis following the final cycle of the qRT-PCR and 2% agarose gel electrophoresis was used to check the specificity of the PCR amplification. The *18S rRNA* gene was included as an internal control [43]. The expression of each gene was normalized against *18S rRNA* by using the 2^−Δ*C*t^ method, and then, the expression of a specific member in a specific sample, as indicated in the legend of each figure, served as a calibrator (1.0); the expression of the remaining genes was expressed as a multiple of the calibrator.

**Table 5:** Real-time PCR primers, annealing temperatures and expected amplicon sizes of six selected *PpySDH* genes.

Gene	Primers	Annealing Temperature (°C)	Amplicon Size (bp)
*PpySDH2*	F 5′-GTCCGTTCCACTGTATGGTT-3′	58	115
	R 5′-GCAAAGGAGTGGAGGAGTC-3′	58	
*PpySDH4*	F 5′-GTTGATGTACAGAGACCATTG-3′	58	115
	R 5′-GCATACGTACGCACACAATTAT-3′	58	
*PpySDH8*	F 5′-GGTGGAAGAAGCCTTTGAAA-3′	58	241
	R 5′-TTACAAGAAGCGGAGGGTTT-3′	58	
*PpySDH12*	F 5′-CCAGTGTAGTATAGCTTCCC-3′	58	135
	R 5′-AGGCCAACAGACTCGTGTC-3′	58	
*PpySDH13*	F 5′-ATTAGAATGTAGAGAAAGGGA-3′	58	175
	R 5′-TTTGGTTCAATAGCCACCC-3′	58	
*PpySDH14*	F 5′-AGGTCATCACAATAAGCACG-3′	58	91
	R 5′-AAACAAACAGAACGAGAAGCC-3′	58	
*18S rRNA*	F 5′-CATGGCCGTTCTTAGTTGGTGGAG-3′	58	110
	R 5′-AAGAAGCTGGCCGCGAAGGGATAC-3′	58	

### 4.7. Statistical Analysis

A completely randomized design was used in the experiment. Standard errors (SE) were used in the data description. The significant differences were determined based using ANOVA, with a significance level of 5%.

## 5. Conclusions

RNA-seq of fruit tissues of a sand pear was carried out, and the expression of 14 *PpySDHs* was identified. Comparative phylogenetic analysis of these *PpySDHs* with other plants supported a close relationship of sand pear with Chinese white pear (*P. bretschneideri*). The expression of *SDH* members varied greatly, with the strongest six accounting for 96% of total transcript abundance. Differences in the expression of six *PpySDHs* during fruit development were observed and were suggested to be related to the divergence in content and composition of sugars accumulated in “Cuiguan” and “Cuiyu” sand pears. The study could lead to a better understanding of the molecular mechanism for sugar metabolism and accumulation in pear fruit.

## Supplementary Materials

Supplementary materials can be found at http://www.mdpi.com/1422-0067/16/06/13065/s1.

## Abbreviations

DAFB: days after full bloom
FW: fresh weight
MDR: medium-chain dehydrogenase/reductase
qRT-PCR: quantitative reverse transcription PCR
RPKM: reads per kilo-base per million
SDH: sorbitol dehydrogenase
SSC: soluble solids content
TPM: transcripts per million
