# Supplementary material for: Genome-Wide Analysis of Sorbitol Dehydrogenase (SDH) Genes and Their Differential Expression in Two Sand Pear (Pyrus pyrifolia) Fruits
Source: Int J Mol Sci. 2015 Jun 9;16(6):13065–83. doi: 10.3390/ijms160613065 (PMC4490486; doi:10.3390/ijms160613065)
Supplement: Supplementary file 1 [file ijms-16-13065-s001.pdf]

Supplementary Information

Table S1. List of 83 identified *SDH* homologous genes from 12 plant species. "N" means that the gene does not have the indicated domain; "Y" means that the gene has the indicated domain; "D" means that the gene has double copies of the indicated domains; and "I" means that the gene has an incomplete copy of the indicated domain.

Family Name	Species Name	No.	Transcript Name	PLN02702 Domain	COG1063 Domain
Rosaceae	*Pyrus bretschneideri*	1	*Pbr013912.1*	Y	N
		2	*Pbr013913.1*	Y	N
		3	*Pbr013914.1*	Y	N
		4	*Pbr013915.1*	Y	N
		5	*Pbr013916.1*	Y	N
		6	*Pbr013917.1*	Y	N
		7	*Pbr022043.1*	Y	N
		8	*Pbr032770.1*	I	N
		9	*Pbr032772.1*	Y	N
		10	*Pbr032773.1*	Y	N
		11	*Pbr032774.1*	Y	N
		12	*Pbr032775.1*	Y	N
		13	*Pbr032776.1*	Y	N
		14	*Pbr032777.1*	Y	N
		15	*Pbr032778.1*	Y	N
	P. communis	16	*TCONS_00015561*	Y	Y
		17	*TCONS_00015562*	Y	Y
		18	*TCONS_00015564*	Y	Y
		19	*TCONS_00015565*	Y	Y
		20	*TCONS_00015566*	Y	Y
		21	*TCONS_00015567*	Y	Y
		22	*TCONS_00015568*	Y	Y
		23	*TCONS_00015569*	Y	Y
		24	*TCONS_00015570*	Y	Y
		25	*TCONS_00015571*	Y	Y
		26	*TCONS_00020677*	Y	Y
		27	*TCONS_00020678*	Y	Y
		28	*TCONS_00020679*	Y	Y
		29	*TCONS_00020680*	Y	Y
		30	*TCONS_00020682*	Y	Y
		31	*TCONS_00020683*	Y	Y
		32	*TCONS_00020685*	Y	Y
		33	*TCONS_00034640*	Y	Y
		34	*TCONS_00034641*	I	N
		35	*TCONS_00034642*	Y	Y
		36	*TCONS_00034643*	Y	Y
		37	*TCONS_00039589*	Y	N

Table S1. *Cont.*

Family Name	Species Name	No.	Transcript Name	PLN02702 Domain	COG1063 Domain
Rosacea	*P. pyrifolia*	38	*PpySDH1*	Y	N
		39	*PpySDH2*	Y	N
		40	*PpySDH3*	Y	N
		41	*PpySDH4*	Y	N
		42	*PpySDH5*	Y	N
		43	*PpySDH6*	Y	N
		44	*PpySDH8*	Y	N
		45	*PpySDH9*	Y	N
		46	*PpySDH10*	Y	N
		47	*PpySDH11*	Y	N
		48	*PpySDH12*	Y	N
		49	*PpySDH13*	Y	N
		50	*PpySDH14*	Y	N
		51	*PpySDH15*	Y	N
	Fragaria vesca	52	*mrna13340.1-v1.0-hybrid*	Y	N
	Malus domestica	53	*MDP0000123910*	Y	N
		54	*MDP0000149907*	Y	N
		55	*MDP0000167088*	Y	N
		56	*MDP0000171573*	Y	N
		57	*MDP0000188052*	Y	N
		58	*MDP0000188054*	Y	N
		59	*MDP0000250546*	Y	N
		60	*MDP0000305455*	Y	N
		61	*MDP0000515106*	Y	N
		62	*MDP0000638442*	Y	N
		63	*MDP0000707567*	Y	N
		64	*MDP0000759646*	Y	N
		65	*MDP0000786110*	Y	N
		66	*MDP0000807470*	Y	N
		67	*MDP0000873573*	Y	N
		68	*MDP0000874667*	Y	N
	Prunus mume	69	*XM_008236083.1*|	Y	N
		70	*XM_008238108.1*|	Y	N
		71	*XM_008238138.1*|	Y	N
	P. persica	72	*EMJ01124*	Y	N
		73	*EMJ15002*	Y	N
		74	*EMJ19358*	Y	N
		75	*EMJ02279*	Y	N
Brassicaceae	*Arabidopsis thaliana*	76	*AT5G51970.1*	Y	N
		77	*AT5G51970.2*	Y	N

Table S1. *Cont.*

Family Name	Species Name	No.	Transcript Name	PLN02702 Domain	COG1063 Domain
Gramineae	*Zea mays*	78	*GRMZM2G175423_T01*	Y	N
		79	*GRMZM2G175423_T02*	Y	N
Rutaceae	*Citrus sinensis*	80	*Csi_CX674141*	Y	N
Solanaceae	*Solanum lycopersicum*	81	*Solyc01g006510.2.1*	Y	N
Vitaceae	*Vitis vinifera*	82	*VIT_16s0100g00290.t01*	D	N
		83	*VIT_16s0100g00300.t01*	D	N

Table S2. Similarity of nucleotide sequences between *PpySDH* genes and corresponding genes in the Chinese white pear reference genome.

Gene Name	Corresponding Gene in Reference Genome	Score	*E*-Value	Identity (%)
PpySDH1	Pbr013912.1	2078	0.0	99
PpySDH2	Pbr013913.1	2023	0.0	99
PpySDH3	Pbr013914.1	1965	0.0	99
PpySDH4	Pbr013915.1	2006	0.0	99
PpySDH5	Pbr013916.1	1978	0.0	99
PpySDH6	Pbr013917.1	1591	0.0	93
PpySDH8	Pbr032771.1	2006	0.0	99
PpySDH9	Pbr032772.1	1290	0.0	94
PpySDH10	Pbr032773.1	1995	0.0	99
PpySDH11	Pbr032774.1	2039	0.0	99
PpySDH12	Pbr032775.1	2028	0.0	99
PpySDH13	Pbr032776.1	1467	0.0	91
PpySDH14	Pbr032777.1	1478	0.0	91
PpySDH15	Pbr032778.1	1701	0.0	95

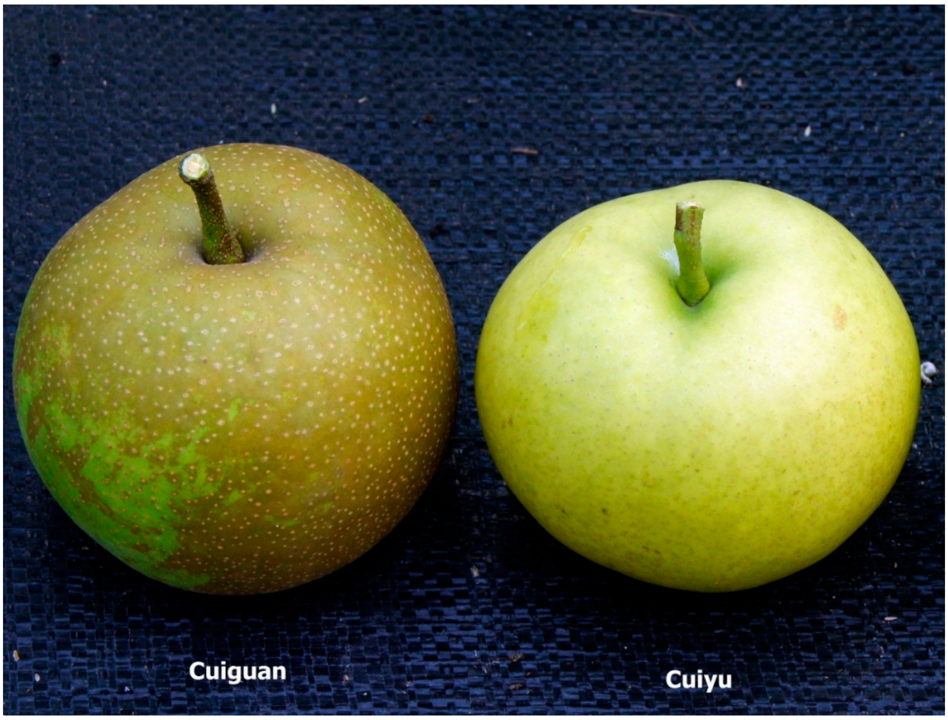

Figure S1. Ripe fruit of”“Cuiguan” and “Cuiyu” pears.

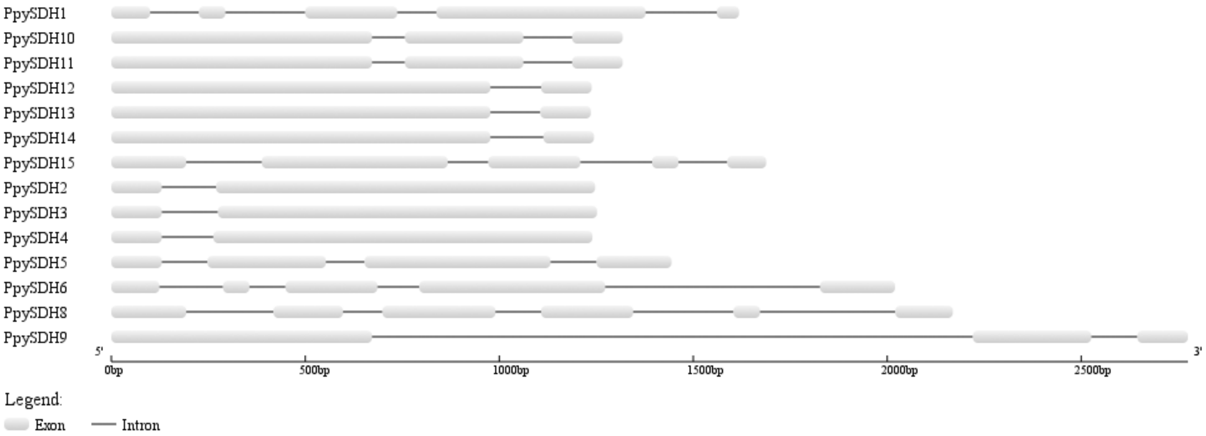

Figure S2. Structure analysis of *PpySDH* genes by using GSDS2.0 (http://gsds.cbi.pku.edu.cn/). The grey rectangles indicate the exons, and the lines indicate the introns.

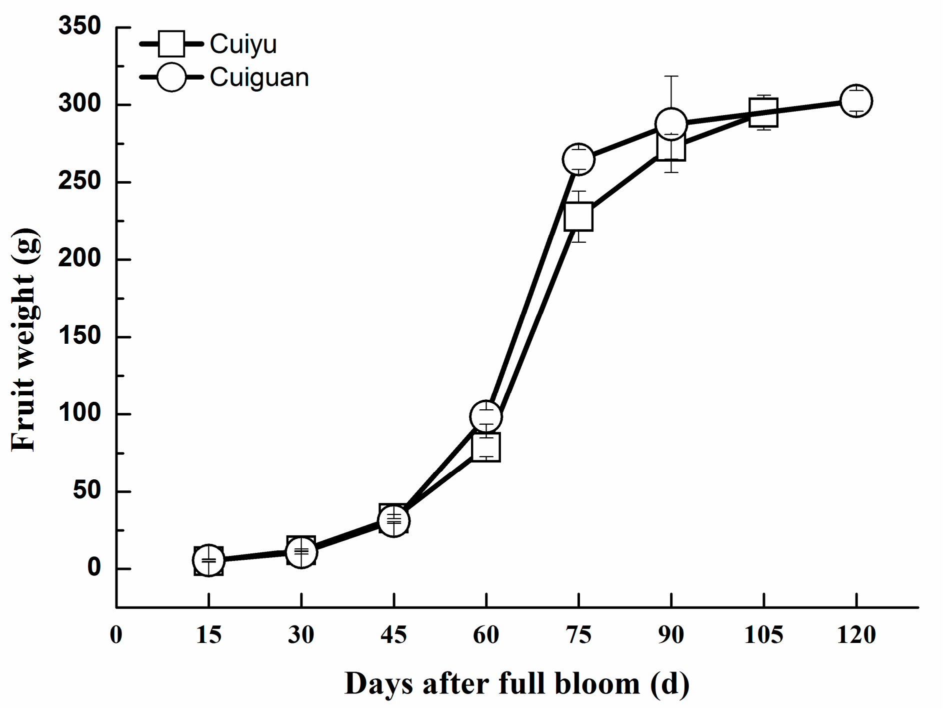

Figure S3. Seasonal changes in average fruit weight of two pear cultivars, “Cuiguan” (-○-) and “Cuiyu” (-□-). The period of “Cuiyu” from flowering to harvest is 105 days, which is 15 days earlier than “Cuiguan”. There is no significant difference in the fruit weight of two cultivars when ripened. Error bars indicate the standard error (SE) from ten biological replicates.
